# Community assessment of tropical tree biomass: challenges and opportunities for REDD+

**DOI:** 10.1186/s13021-015-0028-3

**Published:** 2015-07-25

**Authors:** Ida Theilade, Ervan Rutishauser, Michael K Poulsen

**Affiliations:** 1 Faculty of Science, Institute of Food and Resource Economics, University of Copenhagen, Rolighedsvej 25, 1958 Frederiksberg C, Denmark; 2 CarboForExpert, 1248 Hermance, Switzerland; 3 Nordic Agency for Development and Ecology (NORDECO), Skindergade 23, 1159 Copenhagen K, Denmark

**Keywords:** Community monitoring, Tree biomass, Indonesia, Wood density, Species identification, MRV, REDD+

## Abstract

**Background:**

REDD+ programs rely on accurate forest carbon monitoring. Several REDD+ projects have recently shown that local communities can monitor above ground biomass as well as external professionals, but at lower costs. However, the precision and accuracy of carbon monitoring conducted by local communities have rarely been assessed in the tropics. The aim of this study was to investigate different sources of error in tree biomass measurements conducted by community monitors and determine the effect on biomass estimates. Furthermore, we explored the potential of local ecological knowledge to assess wood density and botanical identification of trees.

**Results:**

Community monitors were able to measure tree DBH accurately, but some large errors were found in girth measurements of large and odd-shaped trees. Monitors with experience from the logging industry performed better than monitors without previous experience. Indeed, only experienced monitors were able to discriminate trees with low wood densities. Local ecological knowledge did not allow consistent tree identification across monitors.

**Conclusion:**

Future REDD+ programmes may benefit from the systematic training of local monitors in tree DBH measurement, with special attention given to large and odd-shaped trees. A better understanding of traditional classification systems and concepts is required for local tree identifications and wood density estimates to become useful in monitoring of biomass and tree diversity.

**Electronic supplementary material:**

The online version of this article (doi:10.1186/s13021-015-0028-3) contains supplementary material, which is available to authorized users.

## Background

Programs aiming at curbing deforestation and forest degradation in tropical regions (REDD+) rely upon cost-efficient techniques to monitor, report and verify forest carbon stocks. A complete enumeration of all living plants in a given landscape is impossible, and most studies rely upon a “sample plot” approach in which all trees are measured. However, the representativeness of a plot network for an entire landscape remains challenging to ascertain [1], but recommendations on the shape, size or number of sample plots have recently been proposed (e.g. [2–4]).

While professional foresters or scientists are generally in charge of establishing such sample plots, several REDD+ projects have recently shown how local communities might represent a cheap and efficient alternative to external professionals [5–7]. In South East Asia, community monitoring was able to measure forest carbon stocks with similar accuracy as that of professional foresters [5]. Error in plot-level biomass estimates carried out by non-professional ranged between ±10% [5, 8]. At plot-level, error in biomass estimates can be divided into: (1) model error, such as the choice of a particular allometric model, prediction errors or error on the model parameters [9, 10], and (2) measurement error on the tree growth variables (e.g. tree diameter or height) or omission of trees. To mitigate these errors, standardized protocols and practices have been developed [11, 12] and generic allometric models to estimate tree biomass are now widely applied.

However, a significant difference in community vs forester’s estimates of biomass (381 vs 449 Mg ha^−1^ respectively) was found in Indonesia by Danielsen and colleagues [5]. This discrepancy is exclusively due to measurement errors, as tree biomass was computed using the same model for both observers. In dense tropical forests, errors of measurement may be due to the presence of buttresses, irregular-shaped trunks, misplacement of the tape measure on the trunk, misreading of the actual measure or error of transcription on the tally sheet. Most REDD+ pilot programs use temporary sample plots to assess carbon stocks. The lack of repeated measurements prevents the assessment of measurements’ accuracy and precision. Indeed, tree diameter could be measured accurately (mean of replicates close to the true value), but imprecisely (high variance among replicates), or precisely (low variance of replicates) but inaccurately (e.g. measured with an instrument calibrated with an incorrect standard) [13]. As a consequence, both imprecision or inaccuracy may inflate the uncertainty surrounding tree biomass estimates.

Large tropical trees are known to be more challenging to measure due to large buttresses or odd-shape stems [14], while they account for a large fraction of above-ground biomass [15]. Hence, forests with numerous large trees are more prone to be affected by errors of measurement and to large uncertainties in their biomass estimates. Due to lack of time, data precision and accuracy are barely assessed and reported in forest carbon monitoring. However, assessing main sources of error will help identifying areas where more investment in explanations and training are needed.

Another source of uncertainty relates to tree wood density (WD) that may vary at tree, species and landscape scales [16, 17]. In low accuracy estimation of carbon stocks (Tier 1), WD are approximated by an average regional default value [18, 19]. More sophisticated tree biomass estimates (Tiers 2 and 3) rely upon allometric models based on WD, tree height and diameter at breast height (DBH) [20]. Hence, botanical identification of trees is an important investment for REDD+ activities to accurately estimate tree biomass and monitor biodiversity. Due to the low number of tropical tree taxonomy experts, it has been proposed that para-taxonomists (people who lack formal education, but who are trained to undertake taxonomic tasks) can provide information at a greater rate and at a lower cost compared to expert botanists and conventional approaches [21]. Even though some communities seems to name trees consistently [22, 23], a previous study from Central Kalimantan, Indonesia resulted in poor matching between vernacular names and actual taxa, possibly due to the variety of dialects encountered [24]. On the other hand, wood densities have been found to be relatively homogeneous within Indonesian tree genera [25], and a congruent identification of the common genera by local monitors could replace the use of average WD with genus-specific values and reduce uncertainties in corresponding forest carbon stock estimates.

The present study addresses the following questions:

1. How accurate and precise are tree diameter measurements carried out by community monitors?
2. Does prior experience from logging inventories reduce measurement errors?
3. Is local ecological knowledge useful for tree identifications?
4. How do different sources of error propagate into tree biomass estimates?

## Results

### Source of errors in tree diameter measurements

**Figure 1:**
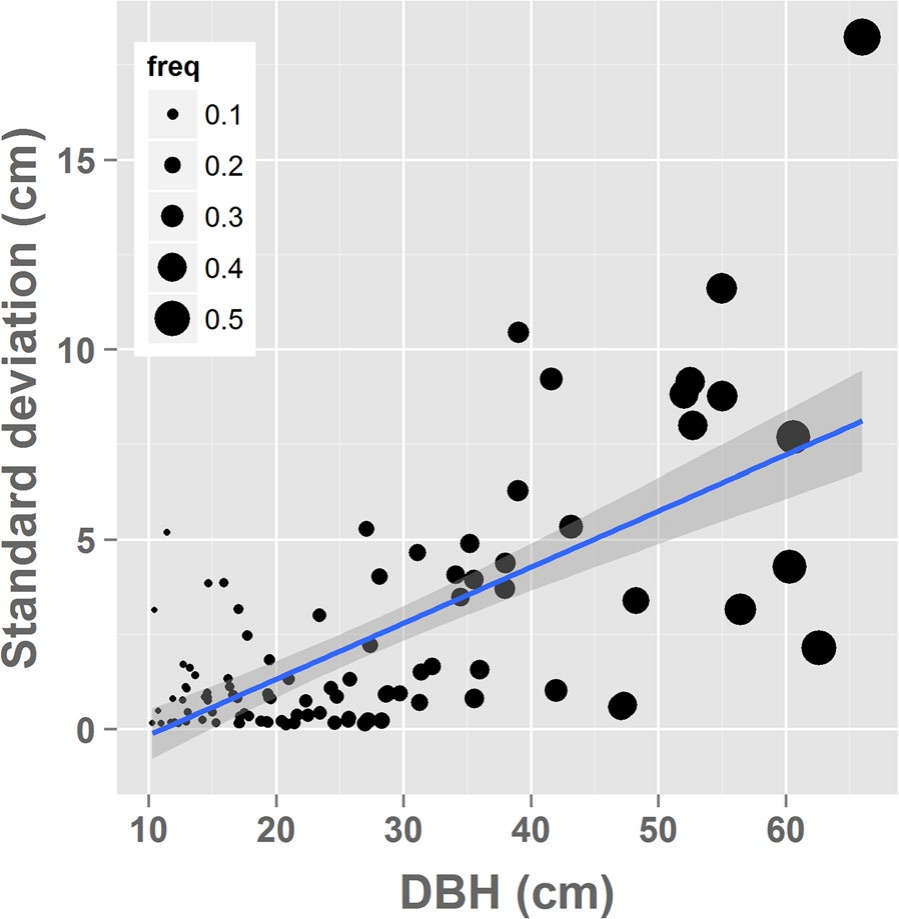
Standard deviation (*Y*-*axis*) around mean DBH measurements (*X*-*axis*). Linear regression (*line*) and 95% CI envelop are shown. *Dot size* is proportional to the frequency of large errors by DBH class.

Tree girth of 103 trees were measured by eleven local monitors, with 95% of all measurements comprised between −5.73 and 5.83 cm around the actual DBH value. Only 86 measurements out of 1,749 felt out of this confidence interval, designated hereafter to as “large errors”. Large errors were more frequent and of greater magnitude (i.e. larger SD) among trees with large DBH (Figure 1). Errors were biased positively, and stand-level biomass was generally overestimated (range −4 to +20%; mean +7%). Half (52.3%) of this errors (|DBH_mes_ − DBH_mean_| > 6 cm) were found among trees designated as having “odd shape” by local monitors, while these trees made up only 16% of the sample. A fifth of the measurements done on trees with odd shape was affected by large errors, significantly more than those carried on more regular stems (16 vs 3% respectively, χ^2^ = 81.3, df = 1, P < 10^−5^).

**Figure 2:**
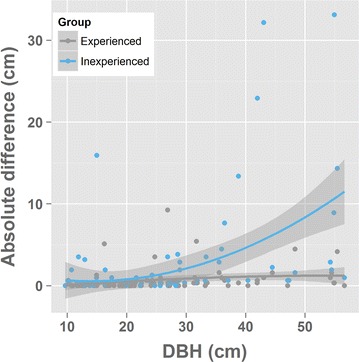
Difference in paired DBH measurements (*Y-axis*) of the same tree DBH (*X-axis*) among experienced (N = 4) and inexperienced (N = 2) monitors. *Smoothed averaged curves* and 95% CI envelop are shown.

Prior experience in measuring trees did not significantly decrease the likelihood of doing a large error (χ^2^ = 2.5, df = 1, P = 0.11). But when the repeatability of measurement was investigated, experienced monitors performed better. Difference in paired DBH measurements significantly differed (Pairwise Student test: t = −2.34, df = 146.4, P = 0.02) among experienced and inexperienced monitors, averaging 0.9 and 2.4 cm respectively (Figure 2).

### Estimating wood hardness

**Figure 3:**
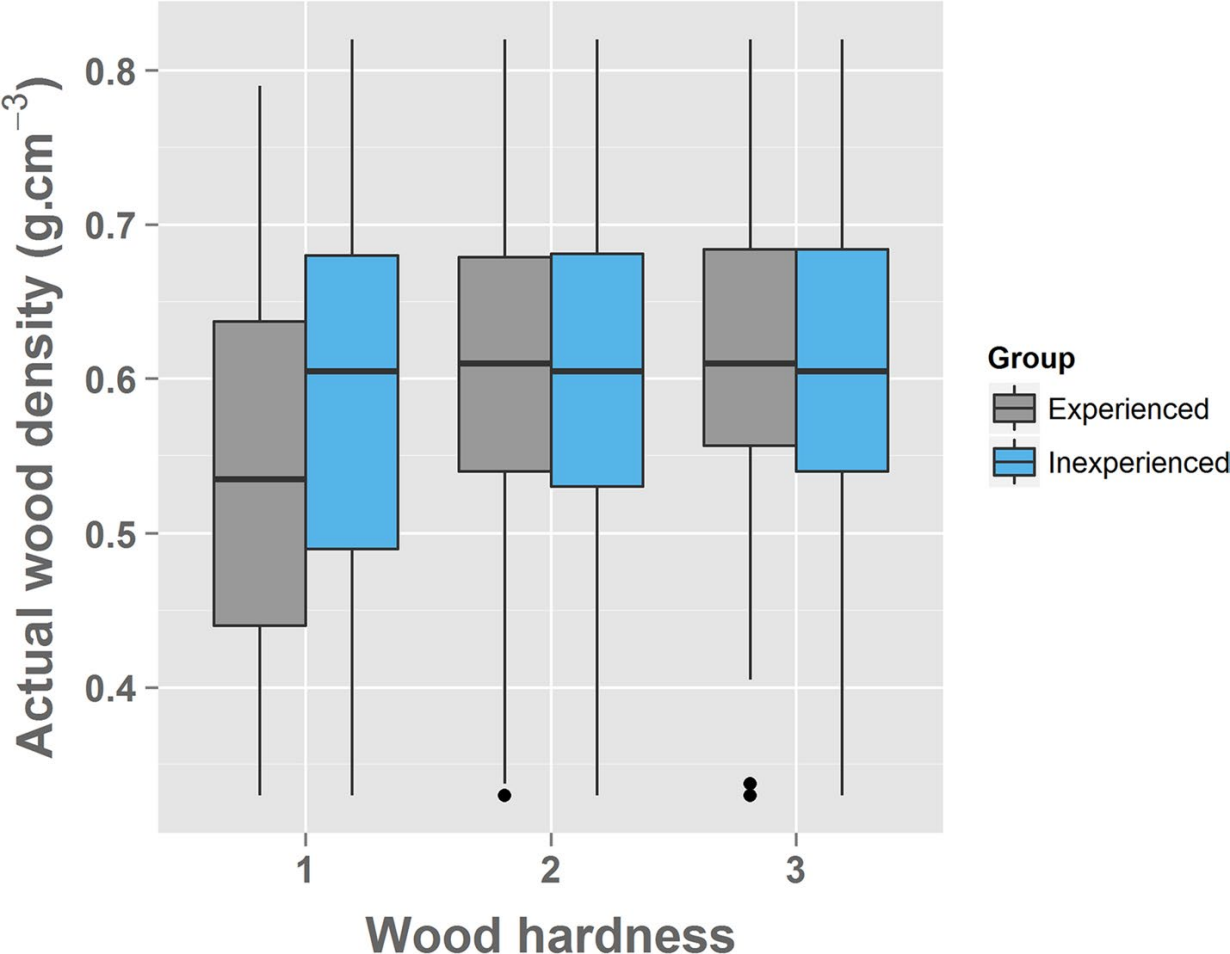
Boxplot of wood densities by wood hardness class estimated by experienced (*grey*) and inexperienced (*blue*) observers.

For each tree, local monitors were also asked to estimate the wood density on a 3-classes scale (i.e. very light, light and heavy). While this simple classification returned generaly poor results (Figure 3), experienced monitors were able to discriminate trees with low wood densities (Figure 3, ANOVA: F_2,613_ = 11.76, P < 10^−4^) while inexperienced monitors could not (ANOVA: F_2,511_ = 0.424, P = 0.655).

### Vernacular identification

**Figure 4:**
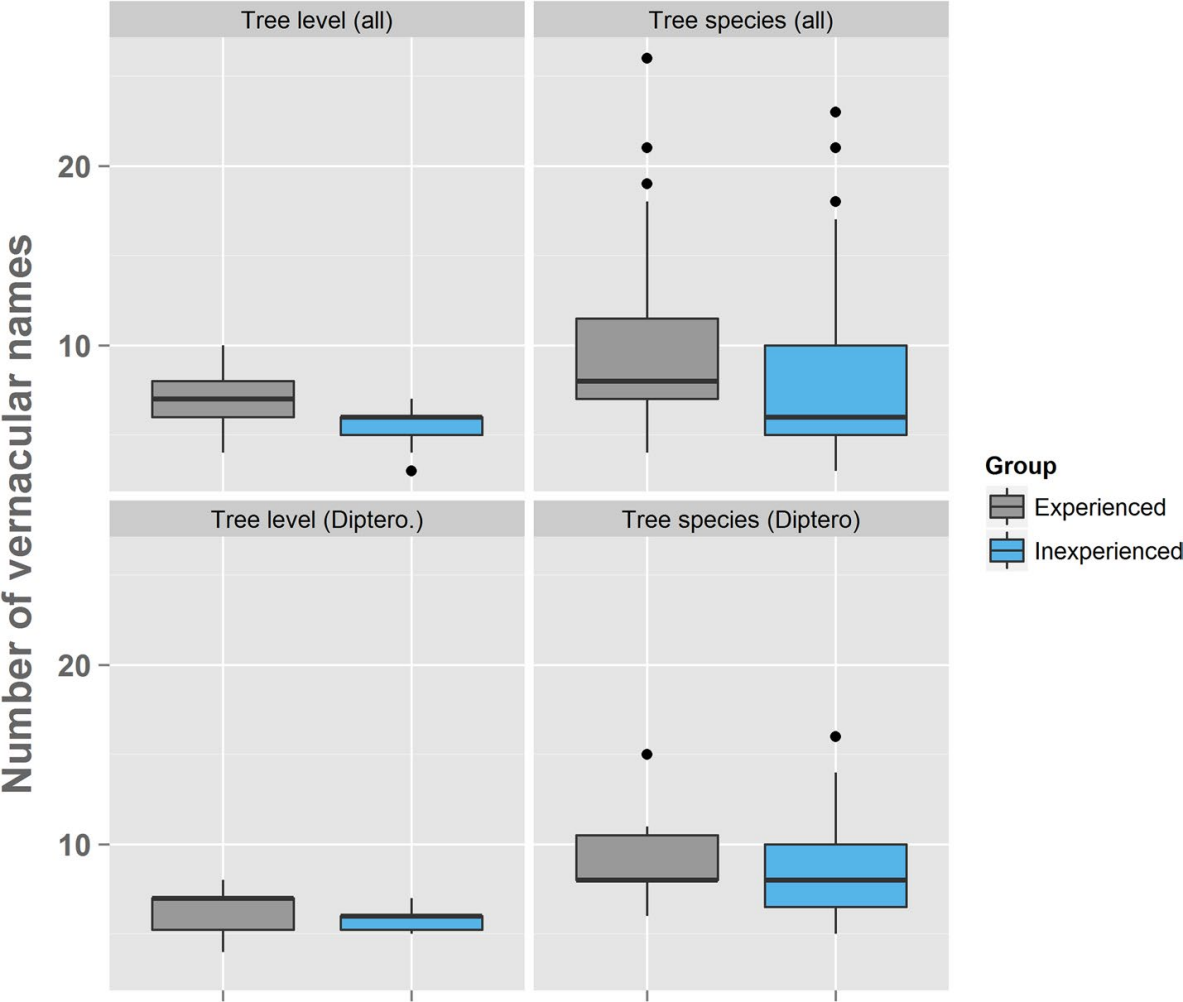
Number of vernacular names (*boxplots*) at tree and species by experienced and inexperienced monitors for all trees (*top*) and Dipterocarps only (*bottom*).

The third information collected in the field was the vernacular name of each tree. Overall, there was very little agreement among observers in naming trees (Figure 4). For instance, the number of vernacular names averaged nine per taxa. More consistency was found among Dipterocarp trees, which were better identified by experienced monitors than inexperienced ones (ANOVA: F_1,42_ = 10.55, P = 0.002).

### Propagating error of DBH measurement and wood hardness into tree biomass estimates

**Figure 5:**
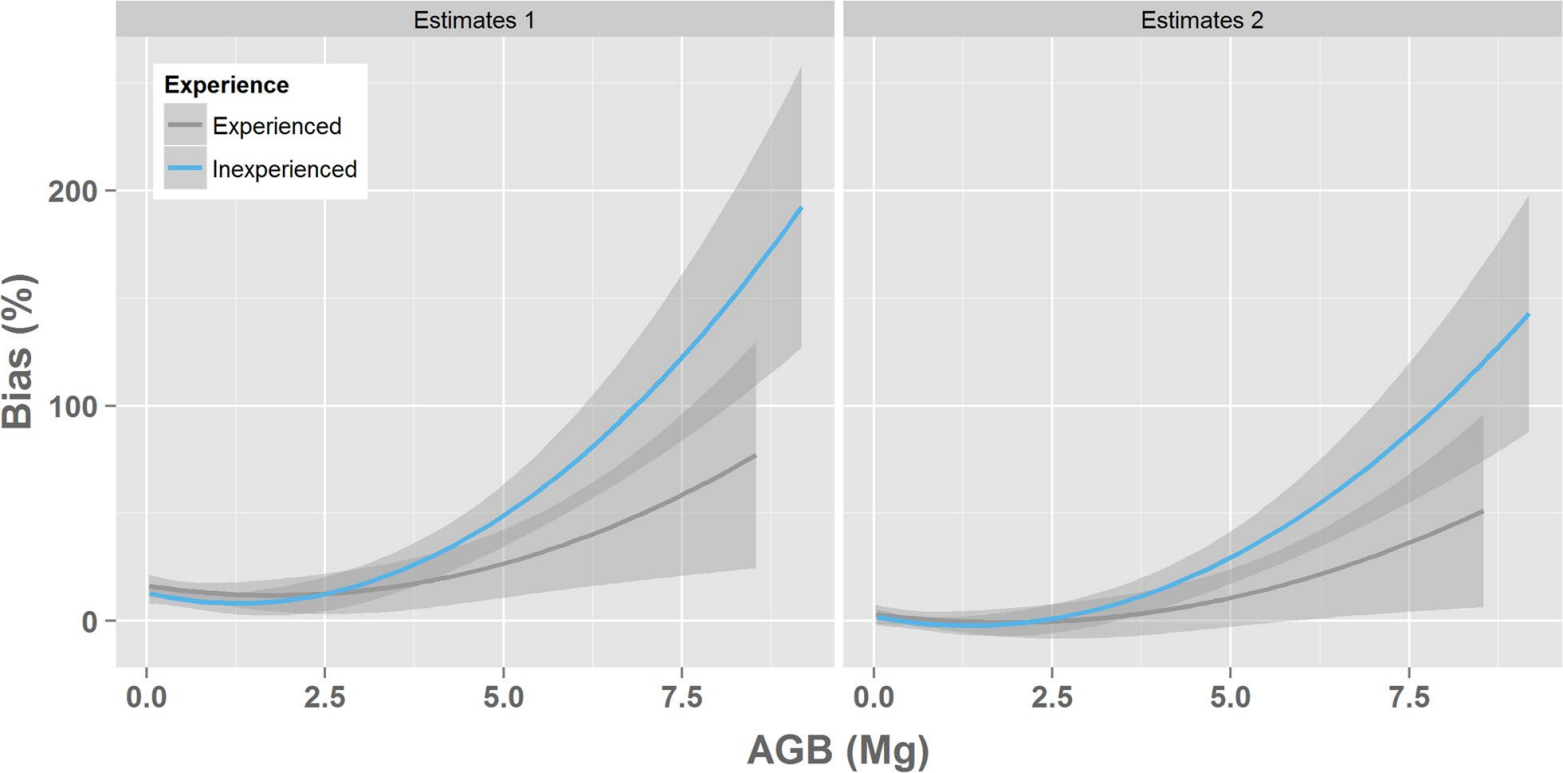
Smoothed average (*line*) and 95% confidence intervals (envelop) difference (%) of tree biomass estimates with (*left*) estimates (*Estimates 1*) computed with wood density derived from wood hardness; (*right*) estimates computed with DBH measurement and default wood density value (0.6 g cm^−3^).

For both experienced and inexperienced monitors, the bias increased with tree biomass (Figure 5). When accounting for DBH measurements and average wood density per wood hardness class, experienced monitors performed better and generated lower bias compared to their inexperienced counterparts (Figure 5, Estimates 1). When all trees were assigned the same wood density, biases lowered but remained high for large trees (Figure 5, Estimates 2).

## Discussion

### Tree diameter measurements

Overall, local monitors had good ability to measure trees, with 95% of the measurements found within 6 cm around the actual DBH. Large errors were not randomly distributed, but increased in frequency (i.e. number of occurence) and magnitude (i.e. breath of SD) with DBH (Figure 1). Half of these errors were found among odd-shaped trees, while these trees made up only 16% of the sample. A fifth of the repeated measures done on odd-shaped trees was affected by at least one large error, significantly more than among regular stems (16 vs 3%). When averaged out at stand level, we found a significant bias towards larger DBH measurements that resulted in an stand-level biomass overestimation of 7%. This error remain low and of similar magnitude as that reported in other studies [25, 26]. We have decided to use the most recent allometric models to calculate tree biomass, as generic models were shown to perform better at our site [27]. However, we acknowledge that the choice of a particular allometric model may result in greater inaccuracies than the physical measurements described above [9].

### Beyond tree measurements

As botanical identification is mandatory to determine specific WD and calculate tree biomass, two methods were tested to see whether local knowledge could help towards this task. The introduction of a simple 3-scales wood hardness classification returned unconvincing results (Figure 3), as inexperienced monitors were not able to distinguish between hardwood classes while experienced monitors were able to distinguish very light wood only. Likewise, more consistency was found among experienced monitors to name Dipterocarp trees (Figure 4), i.e. the main commercial timber family in the region. This is not surprising as their experience consists mainly in identifying commercial hard wood species, including Dipterocarps, during pre-logging inventories [28]. The overall inability of monitors to classify trees based on coarse wood hardness categories may arise from a misunderstanding of this peculiar concept. Local people usually possess sound knowledge on what different species can be used for, including wood properties such as workability, termite resistance, suitability for tools, firewood or boat-making. A possible explanation may lie in their inability to ‘translate’ this knowledge into this simple wood hardness scale. We suggest that future studies take point of departure in emic categories, i.e. categories defined by local people. Overall, there was little agreement among observers in naming trees. This result corroborate a previous study carried in Borneo, where only 10–20% of the vernacular names employed by Dayak para-taxonomists could be related to a given taxa [24]. The great variability in vernacular names in the region is a result of the numerous ethnic groups and dialects encountered in Borneo. Locally, trees are named based on local or traditional usage and names might be restricted to a community or even a group of villagers. Different species or genera having similar properties or usage are often given the same vernacular name. For instance, at our site, some trees were given names that can be translated as “big tree”. Hence collection and interpretation of vernacular names remains challenging. However, vernacular names remain employed in the logging industry and timber trade, but with little consistency with scientific taxonomy [23]. Refining the list of commonly used vernacular names of Bornean trees, and the corresponding botanical identification at species or genus level would improve forest inventories based on vernacular names.

### Improvement of community monitoring in a REDD+ scheme

The discrepancy in forest biomass stocks measured by community monitors and foresters reported in a previous study at our site [5, 28], is likely to be due to the difficulty to accurately measure large trees in dense tropical forests. Measurement errors among odd-shaped trees is recurrent in carbon accounting studies. As tree biomass allometries relate dry mass with a theoretical taper or cylindrical bole diameter, biomass estimation requires tree measurements above any major irregularities of the trunk. Due to the polynomial form of current generic allometric models, a linear relationship between error and DBH (Figure 1) results mechanically in an exponential inflation of uncertainty when expressed in biomass (Figure 5). We have shown that error in biomass estimates inflates with tree biomass and inexperience. For instance, the biomass of a typical tree of 7.5 ton might be over/underestimated by 47 or 80% by an experienced or inexperienced observer respectively (Figure 5, Estimates 2). This difference goes up to 55 and 120% respectively, when estimated WD are included into biomass computation (Figure 5, Estimates 1).

This issue becomes more acute when monitoring forest biomass over time, as rapid radial increments of buttresses will compound the overestimation of biomass increase [29]. While local monitors accurately measured DBH of most trees, much attention and training should be paid on large trees (>60 cm DBH). Prior experience in measuring trees did not lower the likelihood of doing large errors, but increased accuracy of repeated measurements. Thereby, trained monitors are less prone to systematic bias, a key feature in terrestrial carbon monitoring where true biomass value is sought. Accuracy will also be requested to estimate changes in forest carbon stocks over repeated censuses. Indeed, error of measurements and data correction might prevent the detection of any directional change in biomass stock [30].

In a multi-country comparison of the efficiency (i.e. costs and accuracy) of local communities to monitor tree biomass stocks, Brofeldt and collaborators [28] relied at the second census upon a few community members trained initially, while the rest of team received a brief training only. Based on this study, we recommend that all community monitors involved in REDD+ programmes receive a complete training on tree measurement with special attention on dealing with large and odd-shaped trees. When multi-census has to be carried out, points of measurements should be clearly marked in the field (i.e. paint mark on the trunk). Technical improvements to increase accuracy of community-based measurements of carbon stock will likely facilitate the uptake and scaling up of local information as part of the national forest monitoring system (NFMS) and the associated monitoring, reporting, and verification (MRV) system for REDD+ [31]. This is in line with current United Nations Framework Convention on Climate Change (UNFCCC) texts and guidance documents on the technical aspects of REDD+ which outline explicit roles for indigenous people and local communities in implementing REDD+ [32–34].

## Conclusion

Several REDD+ studies have recently shown how community monitors represent a cost-efficient and reliable alternative to external professionals. In this study, we have investigated different sources of error in tree diameter measurements conducted by community monitors and propagated those at both tree and stand levels biomass estimates.

Local monitors had good ability to measure tree DBH with 95% of all measurements found within a confidence interval of 6 cm around the actual DBH. Large errors were more frequent and of greater magnitude among trees with a large DBH (>60 cm DBH) and odd-shaped trunks. Monitors with experience from logging inventories performed better and generated lower bias compared to inexperienced monitors although the likelihood of large errors was identical among both groups. Overall, we found a directional bias towards overestimated DBH among monitors that led to a slight inflation of stand-level biomass (7%).

We suggest that future REDD+ programmes may benefit from the systematic training of local monitors in measuring tree DBH with special attention given to large and odd-shaped trees. A better understanding of traditional classification systems and concepts, possibly combined with a basic training of local monitors in taxonomy, is required for tree identifications to become useful in monitoring either forest biomass, or tree diversity.

## Methods

### Study site and community monitors

The study area is located in the district of Kutai Barat District, East Kalimantan, Indonesia. Monitoring plots were established in the customary forest surrounding the Dayak village of Batu Majang. The tropical lowland rainforest at 300 m.a.s.l. is characterised by species of the Dipterocarp family such as *Shorea* sp., *Dipterocarpus* sp., *Anisoptera* sp., and *Hopea* sp. among other high quality timber species. Despite the customary harvest of a few trees and other non-wood forest products, the forest structure is similar to that of a primary forest. The local community is committed to conserve the forest for various reasons, such as protecting the watershed and hunting/harvesting resources. Several permanent forest plots were established in 2012, in which all trees >10 cm DBH were tagged, measured and identified to species level [27].

Representatives of the local Dayak community helped select eleven participants (referred hereafter to as community monitors) based on their interest and experience with forest resources, to measure the girth, estimate wood density, and identify trees in the permanent plots. All community monitors were male, had attended primary school, and received 3 h of specific training on tree measurement in the field. Six monitors had a prior employment in timber companies, doing surveys (i.e. mapping harvestable stems) for logging operations. This group is referred to as “experienced”, while others (n = 5) with no previous experience are referred to as “inexperienced”.

### Data collected

In 2014, 103 trees were randomly chosen among two permanent monitoring plots and measured by local monitors. While creating a tree-walk and numbering the trees, the community monitors were trained at measuring tree girth and estimate wood hardness. Girth measurement was done at 130 cm height using classical tapes with centimeter units. Monitors were instructed carefully to avoid common mistakes such as a twisted or lax tape, a thumb placed under the tape, and measuring below breast height.

When measurement was hampered by the presence of buttresses, lianas, or trunk deformities, i.e. extra efforts had to be made to measure tree, monitors were asked to record the tree as “odd shaped”. Wood properties of common tree species is often known by local communities. To test whether such information could be used to refine tree biomass estimates, each monitor was asked to assess wood hardness using a simple classification: “1” for very light wood, “2” for floater (light wood) and “3” for sinker (heavy wood). These categories are used in the logging industry and are well-known to local people. Finally, monitors were asked to name each tree using Dayak common names. Community members worked in teams of two people, monitor A measuring the girth, assessing wood density and naming trees along the full tree-walk, and monitor B writing down information on a pre-prepared form.

### Statistical analysis

#### Overall precision

We investigated the distribution of error measurements on a per-tree basis. As each tree was measured at least once by the different community members, we computed the differences between each measurement and the average DBH for each tree. We further used the 5th and 95th percentiles of these differences to identify large errors. For each tree, we defined the actual DBH (DBH_mean_), as the average of all measurements comprised within the 5th and 95th percentiles. The minimum number of measurements used to compute the actual DBH is 12 (max = 17).

The precision of measurements of a given tree diameter refers to the variance of the different measurements. We used the standard deviation to estimate how the different measures spread out from the mean value. The bigger the error, the larger the standard deviation.

$$SD \left( \sigma \right) = \sqrt {\frac{1}{n} \mathop \sum \nolimits \left( {DBH_{mes, i } - DBH_{mean,i} } \right)^{2} }$$

#### Repeatability of measurement

102 trees were measured twice by six observers. We estimated the repeatability of girth measurements among those observers, by calculating the absolute difference among both measurements.

#### Comparison of wood hardness and botanical estimation

In 2012, all trees were identified at species level by a professional botanist [27]. Trees were identified directly in the field to the lowest taxonomical level. Among the 102 trees accounted for in the present study, 70% were identified at species level and 30% at genus level (Additional file 1). From these identifications, wood densities were extracted from the Global Wood Density Database [35] and considered as actual wood densities (WD). The capacity of local observers to group trees in three classes of wood hardness was further assessed with a one-way ANOVA by wood hardness classes and observers experience.

#### Error propagation in tree biomass estimates

We integrated information gathered in the field by local monitors (i.e. wood hardness and DBH measurements) into biomass estimates. Wood hardness was associated to the 25, 50 and 75th percentile of actual wood densities respectively (1 = 0.55, 2 = 0.63, 3 = 0.73 g cm^−3^). Tree biomass (Estimates 1) was computed using a generic allometric model [20], as follow:

$${\text{AGB}}_{\text{est}} = { \exp }\left[ { - 1. 80 3 { } - \, 0. 9 7 6\times {\text{E }} + \, 0. 9 7 6\times { \ln }\left( {\text{WD}} \right) \, + { 2}. 6 7 3\times { \ln }\left( {\text{DBH}} \right) \, - \, 0.0 2 9 9\times { \ln }\left( {\text{DBH}} \right)^{ 2} } \right]$$

where E is a synthetic index of temperature seasonality, maximum climatological water deficit, and precipitation seasonality (E = −0.09162301 at our site), WD is the wood density (g cm^−3^), and DBH, the diameter at breath height (cm).

Alternatively, tree biomass (Estimates 2) was computed using a default WD value for Bornean forests (WD = 0.6, 37) to estimate a “Tier 1” level of uncertainty. Both estimates were further compared to the best tree biomass estimate (AGB_0_), computed with actual WD and DBH (DBH_mean_) as recommended by Tier 3 standard [19]. Differences in tree biomass are expressed as bias (e.g. [estimate_1_ − AGB_0_]/AGB_0_). To check if errors could cancel each other at stand level (i.e. no directional bias), tree biomass were summed for each monitor and the relative bias (%) per monitor was computed as follow:

$$bias_{j} \left( \% \right) = \frac{{\mathop \sum \nolimits AGB_{ij} - \mathop \sum \nolimits AGB_{0} }}{{\mathop \sum \nolimits AGB_{0} }},$$

where *i* = the *i*th tree, *j* = the *j*th monitor and AGB_0_ = best tree biomass estimate.

## Additional file

Additional file 1:
**Table S1.** List of local names commonly used by Dayaks in Batu Majang, Kutai Barat, East Kalimantan, Indonesia.

## References

[1] Chave J, Condit R, Aguilar S, Hernandez A, Lao S, Perez R (2004). Error propagation and scaling for tropical forest
biomass estimates. Philos Trans R Soc B Biol Sci.

[2] Baraloto C, Molto Q, Rabaud S, Hérault B, Valencia R, Blanc L (2013). Rapid simultaneous estimation of aboveground biomass
and tree diversity across neotropical forests: a comparison of field inventory
methods. Biotropica.

[3] Wagner F, Rutishauser E, Blanc L, Herault B (2010). Assessing effects of plot size and census interval on
estimates of tropical forest structure and dynamics. Biotropica.

[4] Walker SM, Pearson T, Casarim FM, Harris H, Petrova S, Grais A (2012). Standard operating procedures for terrestrial carbon
measurement.

[5] Danielsen F, Adrian T, Brofeldt S, van Noordwijk M, Poulsen MK, Rahayu S (2013). Community monitoring for REDD+: international promises
and field realities. Ecol Soc.

[6] Larrazábal A, McCall MK, Mwampamba TH, Skutsch M (2012). The role of community carbon monitoring for REDD+: a
review of experiences. Curr Opin Environ Sustain.

[7] Butt N, Slade E, Thompson J, Malhi Y, Riutta T (2013). Quantifying the sampling error in tree census
measurements by volunteers and its effect on carbon stock
estimates. Ecol Appl.

[8] Molto Q, Rossi V, Blanc L (2013). Error propagation in biomass estimation in tropical
forests. Methods Ecol Evol.

[9] Picard N, Boyemba Bosela F, Rossi V (2014) Reducing the error in biomass estimates strongly depends on model selection. Ann For Sci. doi:10.1007/s13595-014-0434-9

[10] GOFC-GOLD (2012). A sourcebook of methods and procedures for monitoring and reporting
anthropogenic greenhouse gas emissions and removals caused by deforestation,
gains and losses of carbon stocks in forests remaining forests, and
forestation.

[11] IPCC (2014) 2013 Revised supplementary methods and good practice guidance arising from the kyoto protocol

[12] Clark DB, Kellner JR (2012). Tropical forest biomass estimation and the fallacy of
misplaced concreteness. J Veg Sci.

[13] Clark DA (2002). Are tropical forests an important carbon sink?
Reanalysis of the long-term plot data. Ecol Appl.

[14] Slik J, Paoli G, McGuire K, Amaral I, Barroso J, Bastian M (2013). Large trees drive forest aboveground biomass variation
in moist lowland forests across the tropics. Glob Ecol Biogeogr.

[15] Chave J, Muller-Landau HC, Baker TR, Easdale TA, Ter Steege H, Webb CO (2006). Regional and phylogenetic variation of wood density
across 2,456 neotropical tree species. Ecol Appl.

[16] Henry M, Besnard A, Asante W, Eshun J, Adu-Bredu S, Valentini R (2010). Wood density, phytomass variations within and among
trees, and allometric equations in a tropical rainforest of
Africa. For Ecol Manag.

[17] Brown S (1997). Estimating biomass and biomass change of tropical forests: A primer.
FAO Forestry Paper.

[18] IPCC (2006). Guidelines for national greenhouse gas inventories.

[19] Chave J, Réjou-Méchain M, Búrquez A, Chidumayo E, Colgan MS, Delitti WB (2014). Improved allometric models to estimate the aboveground
biomass of tropical trees. Glob Chang Biol.

[20] Sheil D, Lawrence A (2004). Tropical biologists, local people and conservation:
new opportunities for collaboration. Trends Ecol Evol.

[21] Jinxiu W, Hongmao L, Huabin H, Lei G (2004). Participatory approach for rapid assessment of plant
diversity through a folk classification system in a tropical rainforest: case
study in Xishuangbanna, China. Conserv Biol.

[22] de Lacerda AEB, Nimmo ER (2010). Can we really manage tropical forests without knowing
the species within? Getting back to the basics of forest management through
taxonomy. For Ecol Manag.

[23] Wilkie P, Saridan A (1999). The limitations of vernacular names in an inventory
study, Central Kalimantan, Indonesia. Biodivers Conserv.

[24] Slik JWF (2006). Estimating species-specific wood density from the
genus average in Indonesian trees. J Trop Ecol.

[25] Venter M, Venter O, Edwards W, Bird MI (2015). Validating community-led forest biomass
assessments. PLoS One.

[26] Butt N, Epps K, Overman H, Iwamura T, Fragoso JMV (2015). Assessing carbon stocks using indigenous peoples’
field measurements in Amazonian Guyana. For Ecol Manag.

[27] Rutishauser E, Noor’an F, Laumonier Y, Halperin J, Rufi’ie, Hergoualch K, Verchot L (2013). Generic allometric models including height best
estimate forest biomass and carbon stocks in Indonesia. For Ecol Manag.

[28] Brofeldt S, Theilade I, Burgess ND, Danielsen F, Poulsen MK, Adrian T (2014). Community monitoring of carbon stocks for REDD+: does
accuracy and cost change over time?. Forests.

[29] Sheil D (1995). A critique of permanent plot methods and analysis with
examples from Budongo Forest, Uganda. For Ecol Manag.

[30] Muller-Landau HC, Detto M, Chisholm RA, Hubbell SP, Condit R, Coomes DA, Burslem DFRP, Simonsen WD (2014). Detecting and projecting changes in forest biomass
from plot data. Forests and global change.

[31] Torres A (2014). Potential for integrating community-based monitoring
into REDD+. Forests.

[32] UNFCCC (2011) Framework convention on climate change, subsidiary body for scientific and technological advice (SBSTA), methodological guidance for activities relating to reducing emissions from deforestation and forest degradation and the role of conservation, sustainable management of forests and enhancement of forest carbon stocks in developing countries. Draft conclusions proposed by the Chair, Thirty-fifth session Durban, 28 November to 3 December 2011. UNFCCC, Bonn

[33] UNFCCC (2011). Outcome of the work of the ad hoc working group on long-term
cooperative action under the convention. Draft decision [-/CP.17].

[34] UNFCCC (2009) Methodological guidance for activities relating to reducing emissions from deforestation and forest degradation and the role of conservation, sustainable management of forests and enhancement of forest carbon stocks in developing countries. Decision 4/CP.15, FCCC/CP/2009/11/Add.1. United Nations Framework Convention on Climate Change, Copenhagen

[35] Zanne AE, Lopez-Gonzalez G, Coomes DA, Ilic J, Jansen S, Lewis SL et al (2009) Global wood density database. http://datadryad.org/repo/handle/10255/dryad.235. Accessed 28 May 2013

