# Supplementary material for: Community assessment of tropical tree biomass: challenges and opportunities for REDD+
Source: Carbon Balance Manag. 2015 Jul 25;10:17. doi: 10.1186/s13021-015-0028-3 (PMC4515755; doi:10.1186/s13021-015-0028-3)
Supplement: Additional file 1: — Table S1. List of local names commonly used by Dayaks in Batu Majang, Kutai Barat, East Kalimantan, Indonesia. [file 13021_2015_28_MOESM1_ESM.docx]

Table S1: List of local names commonly used by Dayaks in Batu Majang, Kutai Barat, East Kalimantan, Indonesia.

| **Species (scientific name)** | **Nbr. of local names** | **Most used local name** | **Occurrence of most used local name** | **Other  frequently used local names** |
| --- | --- | --- | --- | --- |
| Adinandra dumosa | 25 | Buah | 4 |  |
| Alseodaphne sp. | 14 | Medang, Nyatoh, Salang | 2 |  |
| Artocarpus anisophyllus | 24 | Aka | 7 |  |
| Artocarpus odoratissimus | 12 | Bute | 3 |  |
| Artocarpus sp. | 21 | Pulut, Puteq | 3 |  |
| Atuna racemosa | 39 | Balaq, Buah, Meranti kuning | 5 |  |
| Beilschmiedia sp. | 29 | Kacang | 8 |  |
| Bhesa robusta | 8 | Balaq | 5 |  |
| Cleistanthus sp. | 12 | Saleng | 4 |  |
| Crudia sp. | 17 | Jambu | 14 | (11 times Buah) |
| Dehaasia sp. | 34 | Jambu, Puteq | 14 | (11 times Buah) |
| Dialium indum | 10 | Buah | 6 |  |
| Dillenia reticulata | 10 | Rengas | 4 |  |
| Dillenia sp. | 9 | Jambu | 5 |  |
| Diospyros sp. | 30 | Saleng | 13 |  |
| Dryobalanops lanceolata | 23 | Buah | 7 |  |
| Dyera costulata | 11 | Buah, Puteq | 4 |  |
| Elaeocarpus beccarii | 16 | Rengas | 2 |  |
| Eugenia heteroclada | 8 | Jambu | 6 |  |
| Eugenia stapfiana | 11 | Aka | 4 |  |
| Galearia fulva | 10 | Aka | 5 |  |
| Gironniera nervosa | 24 | Jambu | 13 | (12 times Buah) |
| Gluta aptera | 11 | Rengas | 4 |  |
| Gymnacranthera farquhariana | 7 | Buah | 6 | (4 times Jambu) |
| Hopea mengerawan | 11 | Meranti | 5 | (3 times Pakat) |
| Hopea sp. | 13 | Meranti | 4 | (3 times Buah) |
| Horsfieldia sp. | 14 | Ambang, Apui, Tengkawang | 2 |  |
| Hydnocarpus sp. | 11 | Buah | 3 |  |
| Knema pallens | 10 | Buah | 5 |  |
| Lithocarpus conocarpus | 9 | Aka | 4 |  |
| Lophopetalum sp. | 9 | Buah, Jambu | 4 |  |
| Macaranga conifera | 10 | Aka, Puteq | 3 |  |
| Macaranga pearsonii | 14 | Ajeng, Aka, Jambu | 2 |  |
| Madhuca sericea | 13 | Baleq, Buah, Jambu, Lumut, Meranti | 2 |  |
| Madhuca sp. | 10 | Baleq, Jambu | 3 |  |
| Moultonianthus leembruggianus | 17 | Jambu | 10 |  |
| Ormosia sp. | 11 | Aka | 4 |  |
| Palaquium beccarianum | 17 | Jambu | 7 |  |
| Polyosma integrifolia | 15 | Beliling lalit | 18 |  |
| Santiria sp. | 20 | Jambu | 6 |  |
| Sarcotheca diversifolia | 7 | Beliling lalit | 7 |  |
| Shorea agamii | 15 | Meranti | 10 |  |
| Shorea atrinervosa | 7 | Meranti | 6 | (5 times Balaq) |
| Shorea beccariana | 15 | Tengkawang | 10 | (5 times Meranti sp. and Puteq) |
| Shorea laevis | 24 | Meranti | 13 |  |
| Shorea leprosula | 12 | Meranti | 16 |  |
| Shorea parvifolia | 17 | Meranti | 38 |  |
| Shorea retusa | 12 | Salang | 3 |  |
| Syzygium lineatum | 14 | Salang | 3 |  |
| Syzygium sp. | 31 | Buah | 6 | (4 times Beliling lalit and Medang) |
| Syzygium tawahense | 26 | Jambu | 8 | (5 times Beliling lalit) |
| Vatica umbonata | 14 | Jambu, Meranti | 3 |  |
| Xanthophyllum sp. | 26 | Bute | 8 | (7 times Salang) |
| Ziziphus angustifolius | 9 | Buah | 7 |  |
